# Epidemiology of skeletal trauma and skull fractures in children younger than 1 year in Shenzhen: a retrospective study of 664 patients

**DOI:** 10.1186/s12891-021-04438-8

**Published:** 2021-06-26

**Authors:** Hansheng Deng, Xin Qiu, Qiru Su, Shuaidan Zeng, Shuai Han, Shicheng Li, Zhiwen Cui, Tianfeng Zhu, Zhu Xiong, Gen Tang, Shengping Tang

**Affiliations:** 1 Department of Pediatric Orthopedics, Shenzhen Children’s Hospital, Guangdong Province Shenzhen, P.R. China; 2 Zunyi Medical University, Zunyi, Guizhou Province P.R. China; 3 China Medical University, Shenyang, Liaoning Province P.R. China

**Keywords:** Epidemiology, fracture, skull fractures, infant

## Abstract

**Background:**

Unintentional injury is one of the top three causes of death for infants. However, the epidemiological studies of skeletal trauma and skull fractures in infants younger than 1 year were poorly understood in China. Therefore, our study aimed to examine accidental and emergency attendance in infants under 1 year. It also tried to determine the prevalence and severity of accident types in infants.

**Methods:**

A retrospective analysis was performed on the demographic characteristics of infants younger than 1 year with skeletal trauma and skull fractures who visited the Shenzhen Children’s Hospital from January 1, 2016 to December 31, 2019. Age, gender, fracture site and type, mechanism of injury, length of visit, length of hospital stay, hospitalization cost, and treatment methods were analyzed.

**Results:**

A total number of 675 fractures in 664 infants were included, the median age was 187days (IQR,90-273days), including 394 males and 270 females. The top three fracture sites were the skull (430 sites, 63.70 %), long bones of the limbs (168 sites, 24.89 %), and clavicle (53 sites, 7.85 %). The top three causes of injury were locomotion injuries (256 cases, 38.55 %), falls or trips from low height (from beds, tables, chairs, etc.) (130 cases, 19.58 %), and birth injuries (97 cases, 14.61 %). The greatest amount of fractures occurred in children 1–28 days of life (d) reached a top of 101 cases, followed by 331–365 days, accounting for 15.21 and 10.24 %, respectively. The number of fractures reached a trough of 29 cases in the 29-60d group (4.37 %). And increased again to 65 cases in the 151-180d group (9.79 %). The proportion remained relatively constant at 9 % in the 181-210d group (9.19 %) and 211-240d group (9.64 %). The interval between injury and visiting our hospital was ≤ 72 h in 554 cases.

**Conclusions:**

Special attention should be given to the demographic characteristics of fractures in infants under 1 year of age, and appropriate outreach should be implemented. For example, health education should be provided to aid in the prevention especially for frequently occurring locomotion injuries, and prompt access to specialist medical care should be recommended for skull fractures, which are prone to delayed treatment. In addition, multidisciplinary collaboration should be implemented in trauma care, while also promoting the establishment of trauma centers in specialist children’s hospitals with a stronger capacity to treat pediatric trauma, and a regional system for pediatric trauma treatment.

## Background

A fracture refers to a break in the integrity and continuity of a bone. Bones can be divided according to their location into 2 parts: appendicular and axial skeleton. Of the 10 million pediatric emergency department visits each year in the United States, musculoskeletal injuries account for about 12 % [1], a large proportion of which are cases of pediatric skeletal trauma. Of these, skull fractures caused by direct impact to the skull have an incidence of 2–20 % [2], which may be accompanied by intracranial injury (ICI); the latter is the leading cause of traumatic death in childhood.

Despite the active implementation of measures to prevent accidental childhood injuries, the overall incidence of fractures is increasing, with the literature reporting that fractures account for 8–25 % of all injuries in children per 1000 children annually [3, 4]. Given the predictability of children’s behavioral and psychomotor development, accidental injuries, despite their nature as sudden events, can be considered a disease with both external causes and internal patterns of development, which can be effectively prevented and controlled by taking appropriate measures [5–7]. Nevertheless, only a handful of epidemiological studies have been conducted on skeletal trauma and skull fractures in infants younger than 1 year. In this study, the clinical data of infants younger than 1 year with skeletal trauma and skull fractures who were admitted to the Shenzhen Children’s Hospital were collected, and a comprehensive analysis was performed on their gender, age, type of fracture, and cause of injury. Our aim was to achieve a preliminary understanding of their epidemiological characteristics, so as to provide a basis for the scientific formulation of preventive and intervention measures, and the establishment of scientific norms for trauma care, thereby improving the standards of trauma care.

## Methods and materials

This study retrospectively analyzed 664 infants under the age of 365 days who visited the Shenzhen Children’s Hospital from January 1, 2016 to December 31, 2019. X-ray, computed tomography (CT) and/or magnetic resonance imaging (MRI) were used to confirm the diagnosis of pediatric skeletal trauma and skull fractures.

Patients were divided into twelve age groups: 1-28d, 29-60d, 61-90d, 91-120d, 121-150d, 151-180d, 181-210d, 211-240d, 241-270d, 271-300d, 301-330d, and 331–365 d.

The causes of injury were classified as: locomotion injuries (the cause of injury was defined as “locomotion related” if injury was due to participation in rolling over, moving around, learning to walk, walking, and toddling); falls or trips from low height (falls from beds, tables, chairs, etc.); birth injuries; falls while being carried; traffic injuries; stroller injuries; crush injuries; door-related injuries; impact injuries; cuts; bites; traction injury; falls from height; and unknown causes of injury.

The interval between patient injury and visiting our hospital was categorized as follows: <6 h, 6-11 h, 12-23 h, 24-47 h, 48-71 h, 72 h-5d, 6d-15d, and > 16d. This study was approved by the ethics committee of the Shenzhen Children’s Hospital.

Data analyses were conducted using IBM SPSS Statistics for Windows Version 21.0 (IBM Corp, Armonk, NY). Quantitative variables are presented as the median and interquartile range (IQR: 25th percentile and 75th percentile) or mean and standard deviation. The Kolmogorov-Smirnov test was used for the data distribution study, categorical variables were compared using the χ^2^ test or Fisher exact test, whereas student t test or Mann-Whitney U test, Pearson correlation coefficient were applied for continuous variables. The confidence interval was calculated at 95 %. P values less than 0.05 were considered significant.

## Results

### Distribution characteristics of gender and age

A total of 664 infants were enrolled in this study, including 394 (59.34 %) males and 270 (40.66 %) females, the median age was 187days (IQR,90-273days). there was no significant age difference between males and females (median age males 181days, females 200days, *P* = 0.053), with a median hospital stay of 4.5 days (IQR,3-10days), a mean hospitalization cost of RMB7746.61 (approximately equal to 998.73EUR), and the median interval from injury to hospital visit of 8 h(IQR,4-24 h). (Table 1)

**Table 1:** Demographic information and hospitalization expenses of 664 children

Parameter	Patients n(%)	P*
Numbers	664	
Median Age(day)	187(IQR,90–273)	0.016
Age class		—
1-28days	101(15.21 %)	
29-60days	29(4.37 %)	
61-90days	36(5.42 %)	
91-120days	31(4.67 %)	
121-150days	56(8.43 %)	
151-180days	65(9.79 %)	
181-210days	61(9.19 %)	
211-240days	64(9.64 %)	
241-270days	52(7.83 %)	
271-300days	46(6.93 %)	
301-330days	55(8.28 %)	
331-365days	68(10.24 %)	
Sex		
Girl	270(40.66 %)	—
Boy	394(59.34 %)	
Hospitalization expenses (RMB)	7746.61	0.962
Average time to hospital(hour)	8 (IQR,4–24)	0.021
Hospital stays (day)	4.5(IQR,3–10)	0.004

^*^(The Kolmogorov-Smirnov test)

**Fig. 1:**
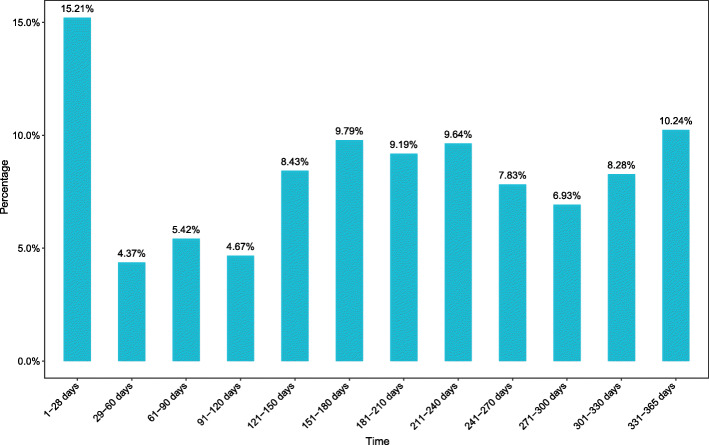
Proportion of the total number of people in each age group This picture illustrates the percentage of children in different age groups in the total

**Fig. 2:**
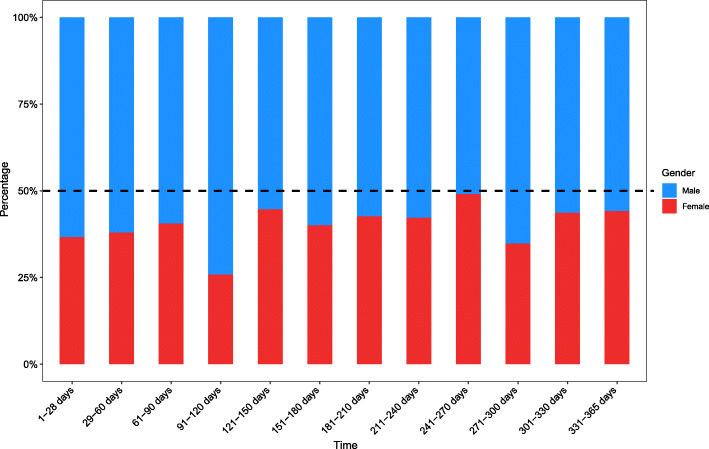
The proportion of male and female children by age group This picture shows the proportion of male and female children in different age groups

**Fig. 3:**
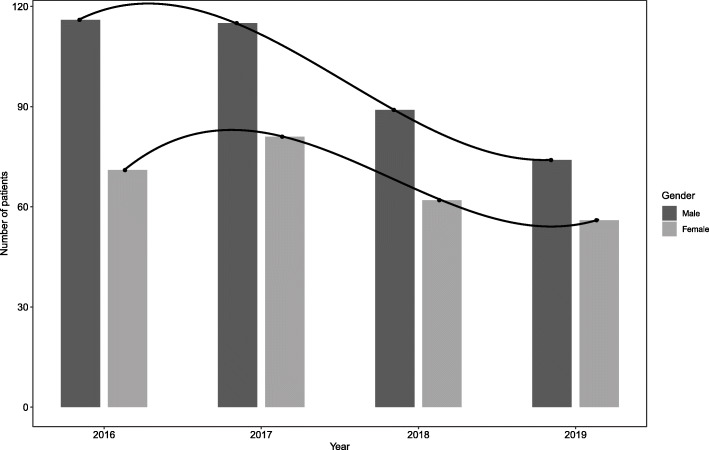
The total number of children changes with the year This picture shows the trend of children of different genders with different years

The number of fractures peaked in the 1-28d group with 101 cases (15.21 %) (Fig. 1), which included 64 males and 37 females (Fig. 2). Among them, the number of fractures caused by birth injuries was 94 cases (93.07 %). Then reached a trough of 29 cases (4.37 %) in the 29-60d group, which included 18 males and 11 females. The number of fractures increased again. The greatest amount of fractures occurred in children 1-28d, followed by 331–365 days, accounting for 15.21 and 10.24 %, respectively. In addition, the proportion of fractures was greater than 9 % in the three age groups of 151-240d which included 111 males and 79 females. In 2016, the total number of fractures reached a peak of 187 cases (28.16 %) (Fig. 3), which included 116 males and 71 females. As the years passed, the total number of fractures showed a decreasing trend (*r*=-0.881, *p* = 0.000), 95 %CI(-0.972,-0. 383), reaching a trough of 130 cases (19.58 %) in 2019, which included 74 males and 56 females.

### Distribution characteristics of etiology

**Fig. 4:**
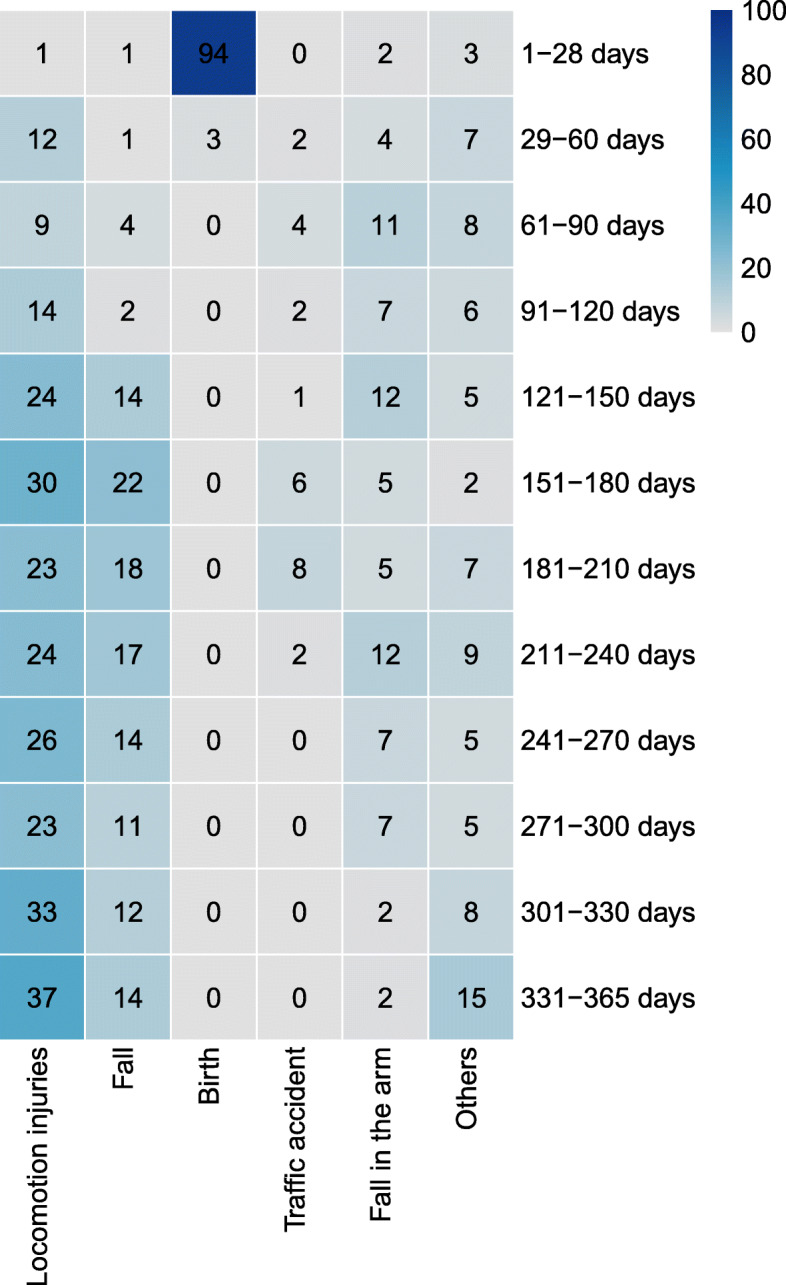
The epidemiology of age group according to different etiologies This picture expresses the distribution characteristics of cause of injury among children of various age groups

The leading causes of pediatric skeletal trauma and skull fractures were locomotion injuries, which included 256 cases (38.55 %) (Fig. 4). The number of locomotion injuries peaked in 2017 then decreased in subsequent years. In addition, the proportion of locomotion injuries was higher in the 331-365d group (37 cases) than in other groups. (Table 2).

**Table 2:** Mechanism of Injury

Cause	N(%)
*locomotion injuries	256(38.55 %)
*fall or trip from low height injury	130(19.58 %)
Birth injuries	97(14.61 %)
Falling from arms of adult	76(11.45 %)
Traffic accident	25(3.77 %)
Stroller fall	20(3.01 %)
Crush injury	13(1.96 %)
Door-related injuries	10(1.51 %)
Impact injury	5(0.75 %)
Cut injury	5(0.75 %)
Bite injury	1(0.15 %)
Traction injury	1(0.15 %)
Fall from height	1(0.15 %)
Unknown	24(3.61 %)

*locomotion injuries include: if injury was due to participation in rolling over, moving around, learning to walk, walking, and toddling, etc.

*Fall injuries include: falls from beds, tables, chairs, etc.

The number of cases with fall or trip from low height injuries was 130 (19.58 %), and the highest proportion of falls was in the 151-180d group (22 cases). There were 97 cases of birth injury (14.61 %), of which the age group of 1–28 days accounted for the largest proportion (94 cases). In addition to birth injury, the main causes of the other injuries are also including locomotion injuries, fall or trip from low height injuries and fall in the arm in this group. There were 76 cases of falling from arms of adult (11.45 %), with the highest numbers in the 151-180d and 211-240d groups. Traffic injuries included 25 cases (3.77 %), with the highest number being in the 181-210d groups.

In addition, there were 24 cases (3.61 %) with unknown causes of injury, 20 cases (3.01 %) of stroller falls, 13 cases (1.96 %) of crush injuries, 10 cases (1.51 %) of door-related injuries, 5 cases (0.75 %) of impact injuries, 5 cases (0.75 %) of cut injuries, 1 case (0.15 %) of bite injury, 1 case (0.15 %) of traction injury, and 1 case (0.15 %) of fall from height.

### Distribution characteristics of fracture sites

**Fig. 5:**
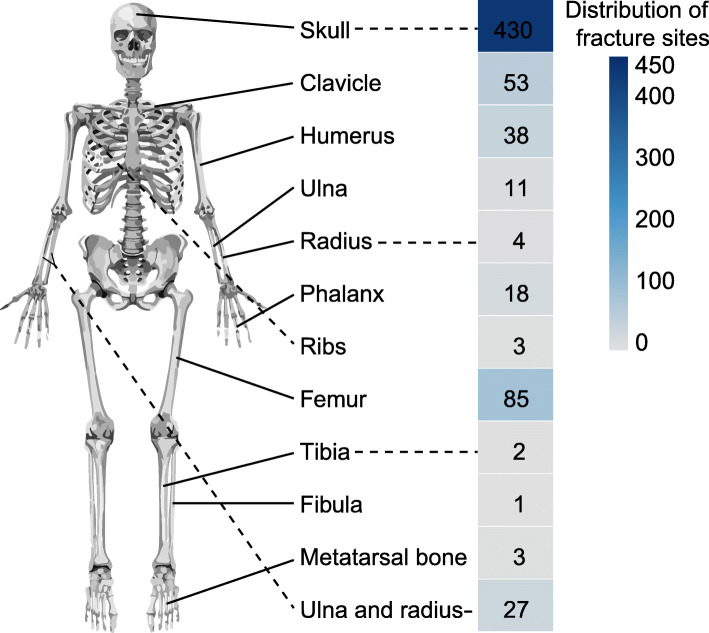
The epidemiology of traumatic fractures according to different age range groups This picture illustrates the distribution characteristics of each fracture site in children of various age groups

**Fig. 6:**
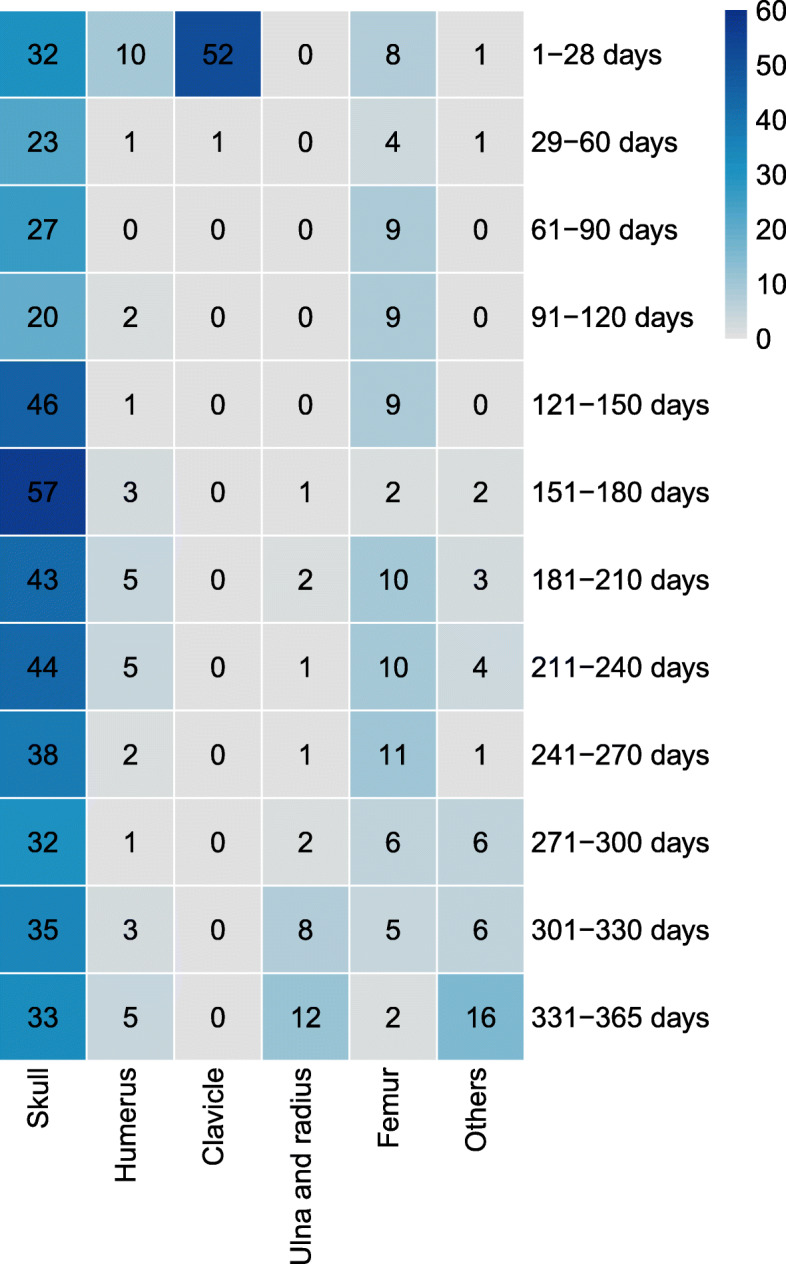
The fracture sites of all patients This picture shows the common fracture sites in all patients

There were 675 fracture sites among 664 infants (Table 3). Among all the fractures, the most common fracture site in both males and females was the skull. The largest number of fractures were skull fractures (430 sites, 63.70 %), with the highest number of skull fractures being in the 151-180d group (57 sites), of which 112 skull fractures (26.05 %) were depressed fractures. In older age groups, there was a decreasing trend (r=-0.103, p = 0.007), 95 %CI(-0.571,-0.026) in the number of skull fractures with increasing age (Fig. 5). In 430 skull fractures, 306 cases (71.16 %) were associated with intracranial injuries. The most common age groups are 121-150d (38 cases), 151-180d (38 cases) followed by 181-210d (28 cases). The common causes of intracranial injuries are locomotion injuries (137cases, 44.77 %), followed by falls or trips from low height (84 cases, 27.45 %). Among the 306 intracranial injury cases, 165 cases were epidural hemorrhage, 111 cases were subdural hemorrhage, 71 cases were subarachnoid hemorrhage, 37 cases were brain contusion and laceration. No infants died due to the intracranial injury. There were 85 femur fractures (12.59 %), which peaked in the 241-270d group (11 sites ,12.94 %). Of the remaining sites of limb fractures, 38 (5.63 %) were of the humerus, 27 (4.00 %) of the ulna and radius, 11 (1.63 %) of the ulna, 4 (0.59 %) of the radius, 2 (0.30 %) of the tibia, 1 (0.15 %) of the fibula, 18 (2.67 %) of the phalanges of the fingers, 3 (0.44 %) of the metatarsal bones, and 3 (0.44 %) of the ribs. There were a total of 53 clavicular fractures (7.85 %), which was the most common fracture in the ≤ 28d group, and included 52 fractures (98.11 %), 51 of which were caused by birth injuries (98.08 %) (Fig. 6).

**Table 3:** Distribution of fracture sites

Site	N(%)
Skull	430(63.70 %)
Femur	85(12.59 %)
Clavicle	53(7.85 %)
Humerus	38(5.63 %)
Ulna and radius	27(4.00 %)
Phalanges of fingers	18(2.67 %)
Ulna	11(1.63 %)
Radius	4(0.59 %)
Metatarsal bones	3(0.44 %)
Ribs	3(0.44 %)
Tibia	2(0.30 %)
Fibula	1(0.15 %)

Among the 675 fractures, 10 were epiphyseal fractures (1.48 %), which included 4 fractures of the humerus, 4 fractures of the femur, 1 fracture of the tibia, and 1 fracture of the phalanges of the fingers. A total of 19 cases had open fractures (2.81 %), which included 16 fractures of the phalanges of the fingers, 2 fractures of the metatarsal bones, and 1 fracture of the skull, respectively. Four cases (0.59 %) were complicated by confirmed nerve injuries, of which 3 had brachial plexus injuries and 1 had radial nerve injury. By reviewing the electronic medical record system, we found two of the three cases of brachial plexus birth injury combined with clavicular fractures, The other case one was combined with humerus fracture.

### Characteristics of the interval between injury and hospital visit

Among the 664 infants with skeletal trauma and skull fractures, the time interval between injury and hospital visit was < 6 h (264 cases, 40.99 %), 6-11 h (113 cases, 17.17 %), 12-23 h (39 cases, 5.87 %), 24-47 h (100, 15.06 %), 48-71 h (38 cases, 5.72 %), 72 h-5d (61 cases, 9.19 %), 6d-15d (44 cases, 6.78 %), and > 16d (5 cases, 0.75 %).(t = 2.896, *p* = 0.023).

For the main reasons of prolonged interval between the injury to our hospital visit > 72 h, among the 110 patients, 48 cases (43.64 %) were due to unsatisfactory treatment in other hospitals; 43 cases (39.09 %) were neglected by their parental initially;14 cases (12.73 %) with other chief complaints were hospitalized and diagnosed by doctors during physical examinations; 2 cases (12.73 %) were a delay in treatment due to the initial misdiagnosis by the diagnosing physician (1.82 %).

Among the 110 infants with an interval between injury and hospital visit > 72 h, 69 cases suffered skull fractures, 20 of which were depressed skull fractures and 19 of which were treated surgically. The causes of injury among infants with depressed skull fractures and delayed hospital visits were locomotion injuries (9 cases, 45 %), falls or trips (4 cases, 20 %) and birth injuries (4 cases, 20 %).

### Characteristics of treatment methods at our hospital

In this study, 440 cases (66.27 %) were treated conservatively and 224 cases (33.73 %) were treated surgically in the all 664 patient cases. Among the infants in the conservative treatment group, 261 cases were males and 179 cases were females. In the 224 were treated surgically, skull fractures were surgically treated in 105 cases (46.88 %), which included 94 cases (89.52 %) who underwent the repair of depressed skull fracture.

Apart from infants treated with cranial surgery, the remaining fracture sites were treated with open reduction (18 cases) and closed reduction (101 cases). Among those treated with closed reduction, 38 (37.62 %) cases received internal fixation with Kirschner wire, 4 cases (0.40 %) were treated with traction, and 59 cases (58.42 %) underwent simple closed and external fixation with plaster cast. The mean operative duration of surgical treatment was (41.76 ± 40.67) min.

Among all infants who underwent surgical treatment, 108 cases (48.21 %) received combined intravenous-inhalation anesthesia with tracheal intubation, 64 cases (28.57 %) received brachial plexus block with intravenous anesthesia, and 52 cases (23.21 %) received intravenous anesthesia.

## Discussion

Infants within the first year of life try to improve their motor skills, learn to how to roll over, sit and walk. Even though Fractures of this ages only accounts for a small proportion in the total number of hospitalized skeletal fracture patients, the proportion of fracture sites, treatments and the causes has its own characteristics. In the present study, we found that: (1) Among the 664 infants included in this study, injuries tended to occur at a younger age and the greatest amount of fractures occurred in children 1-28d, followed by 331-365d, accounting for 15.21 and 10.24 %, respectively; there were more males than females; and conservative treatment was the primary treatment method. (2) The top three fracture sites were the skull, long bones of the limbs, and the clavicle. (3) The leading three causes of injury were locomotion injuries, falling or tripping (falling from a bed, table, chair, etc.), and birth injuries. (4) The interval between injury and hospital visit was ≤ 72 h in 554 cases, > 72 h in 110 infants. The latter included 69 skull fractures and 41 skeletal fractures. The main reason for this delay was the fact that the patients were transferred to our hospital due to unsatisfactory treatment at other hospitals.

Skeletal trauma and skull fractures have specific epidemiological characteristics, which differ between the pediatric and adult populations. The prevalence of fractures among children younger than 1 year is 0.3 %, which is lower than the prevalence reported in other stages of childhood [3, 4]. But for infants whose bones are not fully developed, it can cause serious injuries. Trauma is one of the critical causes of mortality and morbidity among children. [8, 9] In China, epidemiological studies have been conducted on fractures in children of all ages, but fewer studies have focused on infant injuries. Furthermore, some of the epidemiological data are outdated or not updated in a timely manner. Thus, in order to prevent and reduce the harm caused by fractures, it is necessary to investigate the epidemiology of trauma, and hence examining the characteristics of pediatric fractures in this age group can help provide clinical evidence for further prevention guidance.

### Changes in the epidemiology of fractures and etiology in terms of age

Infants under 1-year old experience rapid physical growth and developmental changes. At this stage infants are often exposed to dangerous environments but have not yet developed an awareness of danger of their surrounding environments. In our study, the causes of injury varied significantly by age. The 1-28d group had 101 cases, which accounts for the highest percentage (15.21 %) of overall fracture cases. The main cause of injury in the neonatal stage is birth injury, because the head is usually large, the torso is long, and the ratio of the head to the whole body is 1:4. In addition, the motor system of the neonatal is not fully developed, so there are few sports injuries. The proportion of fracture was the lowest in 29–60 days group. And the number of fractures increased again might because of rapid growth and development of children in this age group, reaching 65 cases in the 151-180d group (9.79 %). And remained relatively constant at 9 % in the 181-210d group (9.19 %) and 211-240d group (9.64 %), in the three age groups, Locomotion injuries, which accounts for 77 cases (40.74 %). When development has reached a peak, and infants are curious about everything around, they roll over, sit, move around, learn to walk, it raises the risk of fractures in terms of motor development.

Our noteworthy finding is that the most common cause of fractures in children was locomotion injuries (38.55 %). The second most common cause of injury was falling from furniture (beds, chairs, tables) (19.58 %), falling from arms of adult accounted for 11.45 %, and stroller-related falls accounted for 3.01 %. Sceats et al. noted that 41 % of accidents among children younger than 1 year could be attributed to falls [9]. Additionally, several studies have documented the risk of falls from beds, chairs [10], prams, changing tables, high-chairs [11, 12], and supermarket trolleys [13]. Similarly, Pollack-Nelson et al. [14] showed that one-third of infant falls are specifically attributed to placing infant car seats on an elevated surface in the home. Studies have also shown that in 53 % of falls related to buggies and prams, the safety harnesses were not being used at the time of the accident, and 5.9 % of such falls resulted in skull fractures [15].

In this study, the most common fracture site was the skull (66.77 %). Claydon et al. [16] noted that minor falls could lead to severe head injuries. Those head injuries are a particular concern due to the malleable nature of infants’ skulls, which predisposes them to skull fractures and intracranial injuries [17]. Mulligan et al[18] found that 32 % of babies with epidural bleeding fell from the bed or sofa. Warrington et al. found that falls occur in 22 % of infants, but the injuries are mild and almost entirely confined to the head, with less than 1 % of falls resulting in serious injury (i.e. concussions and fractures)[19]. However, we found that 71.16 % of skull fractures were associated with intracranial injuries. Our study included 105 infants with skull fractures, who required surgical intervention. Because the infant’s cranial suture is not closed, intracranial injury can occur even in minor trauma even there is no skull fracture. We believe that head CT scanning should be performed in infants with falls, and brain CT imaging is of inestimable value in the early trauma assessment of pediatric patients[20].

In addition, we found that the most common causes of skull fractures in infants were locomotion injuries (49.07 %), followed by falling or tripping (21.63 %). Young children are susceptible to many injury hazards, but have a limited ability to recognize hazards and anticipate the consequences of their actions. Moreover, infants at this age are often unable to protect themselves in the event of a fall.

### Strengthening public health education. The necessity of neonatologists to perform detailed physical examination for neonates

An increase in physical activity accompanies the rapid growth and development of infants younger than 1 year, but lack of hazard awareness and skills to avoid accidental injury. Thus, parents will need to increase their vigilance, and keep infants of this age group away from factors that could lead to injury. Hjern et al. [21]demonstrated that children of young mothers (aged under 24 years) are more likely to be admitted to hospital due to falls. Therefore, adults have a responsibility to protect their children by increasing the appropriate level of supervision[22, 23], and it is especially important to increase the outreach to younger parents. Morrongiello et al. [24] suggested that parental supervision is an essential factor in preventing domestic injuries among young children. Optimal supervision is defined as one where the child remains “visible and accessible” to the caregiver, and consists of three fundamental basic dimensions: attention, proximity, and continuity[25]. The most effective way to prevent injuries in children is to keep them away from relevant hazards. For example, they should be protected during the toddler years by using walkers or carpets in children’s living areas, guard rails can be added around cribs, and safety straps can be used when carrying children. As the infant grows and develops rapidly, the guardian must continually reassess the ability of protective barriers and restraints to ensure the safety of the child.

In this study, the 1-28d group had the highest overall fracture rate with 101 cases (15.21 %), which were mainly for clavicular fractures due to birth injuries. Neonatal clavicular fracture is one of the most common complications of natural birth[26]. This study included 52 cases (51.49 %) of neonatal clavicular fractures are due to birth injuries, of which 47 were normal deliveries, 2 were forceps deliveries and 3 were cesarean deliveries. The most frequently cited risk factors for clavicular fracture due to birth injury were birth weight, shoulder dystocia, forceps delivery, and low Apgar score[27]. The widely accepted hypothesis is that clavicular fracture is due to the impaction of the anterior fetal shoulder against the maternal pubic symphysis[28]. However, in the present study, there were still 3 cases of clavicular fracture due to caesarean section, which suggests the possibility that caesarean section may not prevent the trauma caused by child birth trauma. Neonatal clavicular fractures are not as easy to diagnose, with some studies showing that more than 85 % (46/53) of clavicular fracture cases are diagnosed within 3 days of birth. [26]. We believe that the high-risk children with stable vital signs after birth, should undergo detailed examination by a neonatologist or child health specialist, even confirmation by chest X-ray, given that they show stable vital signs after birth.

The current study on the clinical correlation between clavicle fractures and Brachial Plexus Birth Injury(BPBI) is still in controversial. TOBI’s study found that the incidence of clavicle fractures in BPBI patients was 9.1 %[29], which is much higher than the reported incidences of clavicle fractures at birth of 2.2 and 10.2 per 1000 live births in the general population[30, 31]. However, Oppenheim found that in a series of 21,632 live births, 58 newborns are with clavicle fractures, only 3 of this 58 reference (5 %) had ipsilateral brachial plexus injuries[32]. Same as our study, 52 cases of clavicle fractures caused by birth injuries, only 2 cases had ipsilateral brachial plexus injury (3.8 %). Sever found that clavicle fractures have a protective effect on the brachial plexus which is consistent with our inference[33]. The occurrence of clavicle fractures may help reduce the traction damage to the brachial plexus or cause only slight nerve plexus damage[34]. Because of the limitations of our single hospital data, a multi-center study will be implemented for further explore.

The literature reports that road traffic injuries are the leading cause of infant and child mortality in both developed and developing countries[35]. Car seats (including rear-facing and front-facing car seats, or booster seats) are important protective equipment for infant and children in vehicles, which can significantly reduce the risk of severe injuries and deaths caused by road traffic injury (RTI) [36, 37]. In infants (under 1 year) and toddlers (1–4 years), the correct use of car seats can reduce the risk of death by 71 and 54 %, respectively[38]. It is worth mentioning that in our study, there were 25 cases (3.77 %) who were admitted to hospital due to road accident injuries, with the highest number being in the 181-210d groups, most were due to the lack of child safety seats installed in the car, causing the injuries during a car accident. China’s new traffic regulations now require the use of child safety seats for children under 4 years of age, and children under 12 years of age cannot ride on the passenger side. Furthermore, with the increase use of vehicles, it is necessary to strengthen the implementation of road safety strategies, such as installing speed bumps, improving emergency medical care for traffic accidents, and enhancing road safety enforcement [39]. In addition, we recommend installing special child safety seats in private cars and even in public transport, in order to reduce child mortality and protect children from more serious high-energy severe trauma in the event of traffic accidents.

### The importance of emergency surgeons to improve their knowledge orthopedics and neurosurgery

Data from the US Centers for Disease Control and Prevention show that between 2007 and 2013, the number of emergency department visits for traumatic head injury increased in the 0–4 years and 5–14 years age groups, with a 37.8 % increase in the youngest age group[40]. Research has shown that road traffic accidents are the most common cause of traumatic brain injury in China. The incidence of fall-related traumatic brain injury is expected to increase in the future[41]. Based on the demographic characteristics of pediatric patients presenting with skeletal trauma and skull fractures, we believe that fractures in infants younger than 1 year require more attention than in other age groups. In addition, First-line emergency surgeons should improve their knowledge of orthopedics and neurosurgery, and collaborate with the neonatology and intensive care units in the hospital in a multidisciplinary manner, in order to provide timely and effective care for their patients.

### When skeletal fracture cases occur in children, it should be first examined in specialized children’s hospitals

Most infants with accidental fractures will display behavioral patterns such as crying and screaming, exhibit movement dysfunction in the injured area, and show physical damage visible to the parent. In such cases, most parents are quick to transport the child to a medical center[42]. We recorded the interval between the child’s injury and hospital visit, and found < 6 h: 264 cases, 6-11 h: 114 cases, 12-23 h: 39 cases, 24-47 h: 100 cases, 48-71 h: 38 cases, 72 h-5d: 61 cases, 6d-15d: 44 cases, > 16d: 5 cases. Among children with an interval of > 72 h, there were 69 cases of skull fractures and 41 skeletal fractures, among 69 cases of skull fracture including 20 depressed skull fractures, 19 of which were treated surgically, thus suggesting that skull fractures may be prone to delayed treatment. According to their detailed medical records, the main reason for this prolonged interval in skull fractures was that in 33 cases, the infants were transferred to our hospital from other hospitals due to unsatisfactory results, all of which were non-specialty children’s hospitals. According to the particular developmental status of infants, the treatment plan for adult fractures is not fully applicable to infants whenever possible, children should be first admitted in specialized children’s hospitals or general hospitals with pediatric trauma centers. Some adult specialists should also be trained in pediatric trauma and referral indications, in order to provide the best treatment plan for infants. In addition, a longer interval to hospital visit has been shown to be a significant risk factor for abuse injuries[43]. However, all of our children with prolonged visits have a clear medical history.Their first visit hospital is a non-children specialist hospital, that is the reason why the treatment period has been prolonged instead of being abuse.

Infant in this age group (under 1 year) are always usually closely supervised. However, there were 24 cases in our study which the cause of the injury was unknown, and the chief complaint was that by the time the parents discovered the injury, Moreover, these children had already exhibited significant swelling and functional limitations of the affected limb. Through retrospectively analyzing the electronic medical record system, we did not find that the doctor identified the diagnosis of non-accidental truma(NAT). There are three criteria for the diagnosis of maltreatment injuries are as follows: the number of fractures in the child, the old soft tissue damage in other parts of the body(if or not), the current age-matched weight of children. In these records, we did not find that the doctor made the diagnosis of that the child have Non-accidental trauma. The reason may contribute to our hospital does not have advanced screening mechanisms for NAT and others, the judgement is mainly by the doctor’s physical examination.

In order to accurately monitor the incidence of NAT, it is necessary to establish primary care institutions and carry out population-based surveillance programs. In a study conducted in Wales [44], every child was examined and evaluated every six months before the age of 5 [44]. For the first-time emergency surgeons, the number of fractures, head injury, lower limb injuries, disguise from parents act as the hint for NAT [45–47]. In addition, some studies have identified 9 cited indicators that help distinguish potential abuse injuries from accidental injuries through a literature review[48]. In the later stage, we should carry out research on clinical risk factor prediction models based on these 9 risk factors. If the child has a higher risk factor combined with NAT, the first-time emergency surgeons should discuss with senior staff and/or the Child Protection for more information to avoid missing indicators of NAT. And for hospitals, communities and government functional departments should improve the detection system on abuse to avoid omissions that cause injuries.

### Parents should also raise awareness of fractures in infants

In addition, considering special characteristics of children during this stage, the study also finds out that among the 43 cases (39.09 %) with longer delays in seeking treatment due to parental neglect(the parental neglect refers to parental ignorance, weak safety awareness and inability that prevent infant from relevant hazards) since infants cannot communicate with their parents verbally, which lead most parents do not notice the injury until more obvious signs of injury show – such as swelling of the affected limb and limited limb functions. Also, for cases involving that include head injuries, most of the infants did not exhibit disturbance of consciousness after the accident, without corresponding clinical phenomenon such as convulsions and vomiting, the family members can only noticed their injuries due to the subsequent development of hematoma in the head. The clinical symptoms and severity of skull fractures depend on the nature of the accident that led to the fracture. Depressed skull fractures are caused by strong external forces and often involve the underlying brain tissue, with depressed skull lacerating the dura mater below or penetrating the brain tissue. This type of depressed skull fractures often increases the likelihood of post-traumatic seizures and infections. Hence, among infants with clear history of trauma, parents should take their children to specialized children’s hospital, even in the absence of expected behavioral patterns. Moreover, to prevent the persistence of any potential harm, it is important for parents to raise awareness on the risks of trauma, disseminate basic medical knowledge on the initial management of trauma, and reduce the number of secondary injuries in infants after trauma.

### The establishment of pediatric trauma centers and professional pediatric trauma teams to handle such situations

In the treatment of pediatric trauma, referrals to different levels of pediatric trauma centers should be made based on trauma scores. When trauma centers were established in the US and other developed countries, pediatric trauma did not receive sufficient attention. At that time, there were only a few adult trauma centers and no specialized pediatric trauma centers (PTCs). In the 1970 s, the first batch of PTCs was launched in the US and achieved significant success thanks to the efforts of pioneers such as Kottmeier, Haller, Morse, and others [49, 50]. PTCs have now been established in many parts of the US [51]. However, this has not yet been replicated in China. A separate trauma center can be set up in specialist children’s hospitals, or a relevant pediatric trauma team can be set up in adult trauma centers, with a view to providing efficient pediatric treatment. Pediatric trauma teams should include doctors trained in pediatric emergency medicine, pediatric orthopedics, pediatric surgery and pediatric anesthesiology. This will improve the prognosis of infants and those with more serious injuries. Since 2014, the Shenzhen Children’s Hospital has fully implemented inter-hospital transfer among various hospitals in and around the Shenzhen area (including Dongguan city, Huizhou city, etc.). Within this system, the neonatal inter-hospital transfer team is composed of doctors and nurses with rich experience in rescue from the emergency department, neonatology department and neonatal intensive care unit (NICU). They can provide the best treatment measures for all types of newborns and young children in Shenzhen, including critically ill patients of pediatric trauma.

The PTC includes specialists in emergency medicine, surgery, otolaryngology, ophthalmology, anesthesiology, surgical intensive care unit, radiology, etc. Using a trauma information platform, the PTC can effectively integrate multidisciplinary treatment resources, such as pre-hospital emergency care, emergency department and ICU. The most significant advantage of this approach is that whether it is the information platform, treatment process, or staffing and equipment supply, all of these aspects adhere to the principles of the greatest efficiency and optimal process, which greatly reduces the rescue time. The new model of multidisciplinary joint diagnosis and treatment of children’s traumatic diseases in the PTC utilizes the construction of a trauma treatment system feature pre-hospital care, information sharing and intra-hospital multidisciplinary coordination, which avoids the inadequate coordination among the various disciplines, thereby reducing the disability and mortality rates of infant with trauma, and improving the treatment standards of children with acute and critical trauma.

## Conclusions

For this special group of infants under 1 year of age, attention should be paid to the outreach on pediatric skeletal trauma and skull fractures, with a particular emphasis on health education for frequently occurring locomotion injuries, and prompt access to specialist medical care for skull fractures, which are prone to delayed treatment. Furthermore, we believe that trauma centers should be established in specialist children’s hospitals with a stronger capacity to treat pediatric trauma, which should be combined with the referral cooperation of general hospitals, emergency centers and other specialist children’s hospitals to form a regional system for treating pediatric trauma. This will reduce the mortality and disability rates of children with trauma, thereby positively impacting the protection of children’s lives and health.

## Data Availability

The data sets used and analysed during the current study are available from these corresponding authors on reasonable request.

## Abbreviations

ICI: Intracranial injury
RTI: Road traffic injury
PTCs: Pediatric trauma centers
NICU: Neonatal intensive care unit
NAT: Non-accidental trauma
BPBI: Brachial Plexus Birth Injury
